# Supplementary material for: Patterns of Intron Gain and Loss in Fungi
Source: PLoS Biol. 2004 Nov 30;2(12):e422. doi: 10.1371/journal.pbio.0020422 (PMC532390; doi:10.1371/journal.pbio.0020422)
Supplement: Table S1 — Also available at http://genes.mit.edu/NielsenEtAl/. (4.3 MB ZIP). [file pbio.0020422.st001.zip › NielsenEtAl/html/249.html]

AN0785.1.NCU01213.1.MG07697.1.FG02051.1


```
 CLUSTAL W (1.82) Multiple Sequence Alignments - Introns Inserted


Sequence 1: NCU01213.1	245 aa
Sequence 2: FG02051.1	254 aa
Sequence 3: MG07697.1	213 aa
Sequence 4: AN0785.1	213 aa
Alignment Length: 260 aa
Number Identitical Residues: 100 aa
Alignment Score (without introns) 4960


MG07697.1 	---------------------------------------MASSTT--YKLPELPFAYD~A
NCU01213.1	-------MVNLGS-IWQNLLASQAPLQS----MTGNATTMAGLAT--YSLPQLPYAYN0A
FG02051.1 	MFPLLPLLLLLTSPTSSSSVSAKPDLASDNNILPDNIPTYSAMSVGTYSLPALPYAYD0A
AN0785.1  	------------------------------------------MSTQKYTLPPLPYAYD0A
          	                                           :. .*.** **:**: *

MG07697.1 	LEPHISKQIMELHHSKHHAAYVANLNKALETYASG----QLADRVATLAAINFNAGGHIN
NCU01213.1	LEPYISAQIMELHHSKHHQTYVTNLNNALKVHVAAIASSDIPAQIAQQPAIKFNGGGHIN
FG02051.1 	LEPSISAQIMELHHSKHHQAYVTNLNVALKNYATATSSSDIAGQIALQSAIKFNGGGHIN
AN0785.1  	LEPIISSQIMTLHHQKHHQTYITNLNASLEAQASATQANNVPLLISLQQKIKFNGGGHIN
          	*** ** *** ***.*** :*::*** :*:  .:.  :.::.  ::    *:**.*****

MG07697.1 	HTLFWENLTPASSPDADAATSAPSLVTQIEKQWGSLDQFKAEFSAELLGIKGSGWGWLVK
NCU01213.1	HSLFWKNLAPAETPETNYSKAAPSLAAEIEKTWGSFDEFKKAFSAALLGIQGSGWGWLVK
FG02051.1 	HSLFWENLCPSSSPDSK-PDSAPTLGAEISKTWGSIEAFQETFKKTLLGLQGSGWGWLVK
AN0785.1  	HSLFWENLAPYNSPATDIVASAPELKRAIEGQFGSVEAFIKAFNTVLLGIQGSGWGWLVK
          	*:***:** * .:* :.   :** *   *.  :**.: *   *.  ***::*********

MG07697.1 	EKSAAG-ALVVVTTKDQDSVPSG-IPVFGVDMWEHAYYLQ0YLNGKAAYVENIWKVINWK
NCU01213.1	ESTAEKGRLRIITTKDQDPVVGGEVPVFGVDMWEHAYYLQ0YLNGKAAYVENIWKVINWK
FG02051.1 	DTQG----LRIVTTKDQDPVVGGEVPIFGVDMWEHAYYLQ0YLNGKAAYVDNIWNVINWK
AN0785.1  	DSAG---KLDIVTTKDQDPVTGL-VAIFGVDMWEHAYYLQ~YLNNKAAYVEGVWKVLNWG
          	:. .    * ::******.* .  :.:************* ***.*****:.:*:*:** 

MG07697.1 	TAEKRFASPGRSEVFKALHASL
NCU01213.1	TAEERFQGS-REDAFADLKALL
FG02051.1 	TAEARFTGS-REDAFKVLRASI
AN0785.1  	VAEERFKSGVPIEVSGGGIKL-
          	.** ** .    :.
```
